# From early Earth to Enceladus—mineral electrochemistry could drive organic synthesis

**DOI:** 10.1038/s41467-026-71131-6

**Published:** 2026-04-09

**Authors:** Seneca J. Velling

**Affiliations:** 1 NASA Jet Propulsion Laboratory, California Institute of Technology, Pasadena, CA USA; 2 Division of Engineering and Applied Science, California Institute of Technology, Pasadena, CA USA; 3 Kavli Nanoscience Institute, California Institute of Technology, Pasadena, CA USA

**Keywords:** Biogeochemistry, Astrobiology, Geochemistry, Origin of life, Electrochemistry

## Abstract

Hydrothermal systems in the ocean can behave like fuel cells when minerals connect reduced vent fluids to oxidized seawater, sustaining electrical potentials that drive reactivity. A *Nature Communications* study experimentally demonstrates how that potential could reduce CO_2_ on minerals to form organic products under conditions relevant from the early Earth to Enceladus.

At some point in Earth’s history, chemistry transitioned from abiotic organic synthesis—the building of more complicated organic molecules from smaller ones by nonbiological means—to biochemistry. Hydrothermal settings are compelling sites for the origins of this transition because they are natural chemical reactors. In this non-equilibrium setting; hot, reduced fluids continuously mix with cold, oxidized seawater to precipitate minerals on whose surfaces organic molecules react. We typically frame these surface reactions as affected by temperature, pH, and mineral catalysis, but many laboratory analog vent environments also sustain measurable electrical potentials, with the minerals forming these vents offering a conductive pathway for electrons to flow^1–4^. This matters because electrochemical boundary conditions can influence not just *how fast* reactions proceed, but *which* products form^5,6^. That knowledge is essential for creating lab analogues of early Earth and interpreting data from ocean worlds in our solar system.

Zhong et al. address these questions directly by treating CO_2_ reduction as an electrochemical reaction on *mineral substrates* — specifically, carbonates and layer (phyllo-) silicates^7^. Using mineral-coated electrodes, they activated the otherwise weakly CO_2_-reducing Ca/Mg carbonates by adsorbing trace transition metals (most notably Cu and Zn) onto the mineral surfaces. When an applied negative potential was held (cathodic bias), these adsorbed and incorporated metal ions were reduced on/in the mineral, becoming the immediate catalytic sites for catalysis of organic reactions. Interestingly, the mineral substrate is not a passive support. The data suggest the mineral matrix facilitates proton delivery and water activation at the interface, accelerating hydrogenation steps once CO_2_ is activated at the reactive metal center. The carbonates and clay/silicates act as mineral substrates, hosting Cu/Zn sites and shaping the local chemistry that controls CO_2_ reduction to more complex products. By exploring a broad applied-potential window, Zhong et al. mapped how selectivity for CO_2_-reduction changed for mineral-supported active sites. In natural hydrothermal systems, measured vent potentials and redox gradients overlap portions of the studied voltage range^1–4,8^. Within those overlapping windows this study offers a constraint on what products we may expect to find and where competitive reactions (H_2_ evolution) occur.

## Hydrothermal vents have *potential* for prebiotic chemistry

Redox potentials vary with conditions^4^ (Box 1), so comparing Zhong et al.’s measurements to hydrothermal settings, we need to translate to the language of E_h_-pH-T. Natural fluids often express *mixed* potentials from a multitude of redox-active species rather than a single equilibrium couple^8,9^. In hydrothermal systems, the H^+^/H_2_ couple is frequently used as a reference because H_2_ is both a common reductant in water–rock interactions occurring in these settings and is an electron donor that couples to organic reactions through mineral-mediated electron transfer^3,5^. That is why modestly reducing potentials in one pH–T regime can become substantially more reducing in another^4–6^.

Hydrothermal vents and chimneys can sustain measurable redox potentials and electrical gradients, including signals consistent with current generation across mineral structures in contact with both reduced hydrothermal fluids and oxidized seawater^1–3,10^ — effectively a natural analogue to a fuel cell^11^. Thermodynamic driving forces at vents can be harvested electrochemically, and the measured potentials depend on temperature, pH, and the relevant redox couples in the interacting hydrothermal fluid and ocean^4^. The same has been shown in the lab scale analogues of hydrothermal chimneys, injection chemical gardens^12^. These geo-electrochemical driving forces vary considerably in local magnitude, stability, and by vent type^1,3^.

Box 1 Relating redox potential, pH, and temperature to reducing power in hydrothermal systems Potential measurements in natural waters reflect a mix of many equilibrium couples, while lab studies report a particular redox potential vs. a reference, typically the standard hydrogen electrodes (SHE).

**Nernst equation shifts with T and pH and activities of redox species**

For the reaction ${{{\rm{Ox}}}}+{{{{\rm{n}}}}\cdot {{{\rm{e}}}}}^{-}\rightleftharpoons {{{\rm{Red}}}}$, the Nerst equation tells us at the equilibrium, the potential depends on the ratio of oxidized (Ox) to reduced (Red) species, the temperature (*T*), and the number of electrons (*n*).

$$E={E}^{\circ }+\frac{{RT}}{{nF}}{{{\mathrm{ln}}}}\left(\frac{{a}_{{{{\rm{Ox}}}}}}{{a}_{{{{\rm{Red}}}}}}\right)$$

***Ox/Red ratio:*** A 10x change in ${a}_{{{{\rm{Ox}}}}}$ **/** ${a}_{{{{\rm{Red}}}}}$ shifts *E* by $\Delta E\approx 2.303\,{RT}/{nF}$

***Temperature & number of electrons:*** T and *n* scale linearly — for *n* = 1 electron in a redox reaction, Δ*E* is ~59 mV at room temperature (25 °C), ~75 mV at 100 °C, and ~105 mV at 250 °C^4,9^.

**H**^**+**^**/H**_**2**_ **as a diagnostic reductant**

H_2_ is a common ${{{{\rm{e}}}}}^{-}$ donor produced by water-rock reactions in hydrothermal systems. The reaction $2{{{{\rm{H}}}}}^{+}+2{{{{\rm{e}}}}}^{-}\rightleftharpoons {{{{\rm{H}}}}}_{2}$ provides a convenient way to relate pH and H_2_ to reducing power:

$${E}_{{H}^{+}/{H}_{2}}\approx {E}^{\circ }+\frac{{RT}}{2F}{{\mathrm{ln}}}\left(\frac{{\left[{H}^{+}\right]}^{2}}{{p}_{{H}_{2}}}\right)$$

Increasing pH (lower $[{H}^{+}]$) or higher partial pressure of H_2_ (${p}_{{H}_{2}}$) both make *E* more reducing.

${{\mathbf{e}}}^{{-}}$ **released by oxidizing vent fluids (e.g., H**_**2**_**) can be conducted through minerals to reduce seawater oxidants such as CO**_**2**_**—analogous to a natural fuel cell**^11^.

***At what potentials do geo-electrochemical gradients drive organic reactions?***

(i) pH 10 hydrothermal fluid venting into pH 5.5 seawater at ~250 °C can shifts *E* negative by 100s of millivolts^4^
(ii) Between 25 − 300 °C, from pH 2 to 12, oxidizing even 1 mmol kg^−1^ H_2_ can generate potentials ranging from 0 V to as low as −1.1 V vs. SHE^5,6^.
(iii) Minerals in hydrothermal vents are often much more reducing than the surrounding seawater and can catalyze organic reactions^3^

## Trace metals catalyze best in an active mineral matrix

Zhong et al. identify a simple yet consequential mechanism for expanding organic geochemistry on common seafloor minerals: trace amounts of transition metals can “activate” otherwise weakly CO_2_-reducing (Box 2) carbonates and phyllosilicates, shifting the products away from nearly exclusive H_2_ evolution and toward larger (C_2_+) carbon species^7^. In their experiments, surface-bound Cu and Zn active sites were particularly effective. They identified that the carbonate substrate was actively participating in this process, facilitating the reaction at the trace metal center by dissociating water to generate a supply of protons. Consistently, they observed faster turnover of surface-bound intermediates and hydrogenated products on mineral surfaces (*CHO/*HCHO), relative to pure Cu (Box 2).

Potential does not merely speed reactions up or down but biases the relative rates of competing steps on the mineral’s surface. For CO_2_ reduction, many products lie very close together in potential, so catalyst identity and the local interfacial environment often exert control over products^13,14^. Prior geo-electrochemical and metal catalysis studies have shown that product identity depends strongly on both applied potential and mineral/metal composition under different conditions and catalyst types^6,14–19^. In Zhong et al., product distributions were influenced by mineral-hosted Cu/Zn sites and the host mineral; the potential dependence was inseparable from how the mineral matrix conditions the local environment. They mapped where different products emerged versus where the formation of H_2_ was preferred. In addition, they found that when NH_3_ was present, acetamide formation was observed, showing that under the same electrochemical boundary conditions, CO_2_ reduction can feed directly into C–N bond formation^7^. This framing is particularly useful because it separates two questions that are often conflated: (1) whether a pathway is chemically accessible at all, and (2) whether it could generate products at rates high enough to be measurable and chemically useful in the local environment.

Box 2 CO_2_ reduction in water: forming more complex organics on mineral surfaces Dissolved inorganic carbon exists as an equilibrium mixture whose proportions depend on pH. By coupling to an oxidative reaction, like $2{{{{\rm{H}}}}}^{+}+2{{{{\rm{e}}}}}^{-}\rightleftharpoons {{{{\rm{H}}}}}_{2}$ (Box 1), CO_2_ can be reduced to one-carbon (C_1_) products (CO, formate). Hydrogenation and further reduction to CH_4_ and C_2_+ products requires multi-step surface chemistry on hydrothermal minerals.

**CO**_**2**_ **Reduction in water**

$$\begin{array}{c}{{{{\rm{CO}}}}}_{2({{{\rm{aq}}}})}+{{{{\rm{H}}}}}_{2}{{{\rm{O}}}}\rightleftharpoons {{{{\rm{H}}}}}_{2}{{{{\rm{CO}}}}}_{3}\rightleftharpoons {{{{\rm{HCO}}}}}_{3}^{-}+{{{{\rm{H}}}}}^{+}\rightleftharpoons {{{{\rm{CO}}}}}_{3}^{2-}+2{{{{\rm{H}}}}}^{+}\end{array}$$

**Key point:** “CO₂ reduction” in water occurs within an equilibrium mixture, not a single species. At pH ≥ 7, dissolved inorganic carbon is mostly ${{{{\rm{HCO}}}}}_{3}^{-}$ and ${{{{\rm{CO}}}}}_{3}^{2-}$, while ${{{{\rm{CO}}}}}_{2}$ increases as acidity increases.

***CO and HCOOH are the simplest*** CO_2_ *reduction products*:

$${{{{\rm{CO}}}}}_{2}+2{{{{\rm{H}}}}}^{+}+2{{{{\rm{e}}}}}^{-}\to {{{\rm{CO}}}}+{{{{\rm{H}}}}}_{2}{{{\rm{O}}}}\,{{{\rm{or\; HCOOH}}}}$$

***More complex organics take multiple reaction steps***:

$${{{\rm{C}}}}{{{{\rm{O}}}}}_{2}\to {{{\rm{CO}}}}\to \left\{{{{\rm{CHO}}}},{{{\rm{HCHO}}}},{{{\rm{etc}}}}\right\}\to {{{{\rm{CH}}}}}_{4}\,{{{\rm{or}}}}\,{{{{\rm{C}}}}}_{2} \!+$$

**Mineral surfaces mediate this process**.

Surfaces promote organic reactions by holding intermediates (e.g., CO/CHO/HCHO) in place long and close enough for C–C bonds to form.

Different minerals promote different reactions by altering adsorption strength and local proton availability at their surface.

## Are these reactions plausible in hydrothermal vent settings?

Mapping which potentials select for different chemical pathways offers an ability to anticipate which products should emerge in different local environments, in what amounts, and at what rates. So, are comparable potentials and current densities sustained at mineral surfaces in natural environments, and for long enough to matter for organic synthesis? Evidence for natural current generation and spatially varying potentials exists, but is very system dependent^1,3,4^. Hydrothermal systems already demonstrate that reduced carbon species can be generated and sustained: ultramafic-hosted vents can be methane- and hydrogen-rich, and multiple sites show measurable inventories of low–molecular–weight organics in vent fluids^20^. At the Lost City Hydrothermal Field, for example, vent fluids contain elevated concentrations of formate, acetate, and dissolved organic carbon, providing a natural benchmark for the distribution of carbon products that can be present where strong redox and pH gradients persist^21^.

If generated organics are comparable in natural settings to experiments, this then raises the question: are trace metals available and persistent on relevant mineral substrates under hydrothermal conditions? Trace metal availability depends strongly on temperature, sulfide activity, organic ligands, and precipitation/adsorption processes^22–27^. By loading common carbonates with adsorbed CuCl_2_/ZnCl_2_ on a similar order of magnitude to the ppm-levels of Cu/Zn in the oceanic crust, Zhong et al. offer a compelling comparison to natural environments while leaving open the question of how much of that inventory remains as reduced surface sites for catalysis under vent conditions. That offers an opportunity for others to explore under what geochemical conditions can reduced metal sites be generated, stabilized, and regenerated on natural minerals when in competition with sulfidation/oxidation pathways.

## Broader implications for astrobiology and planetary sciences

Chemistry requires driving forces to build up; the temperature, pH, and redox gradients in hydrothermal vents might have supplied the early Earth with the energy it needed for organic chemistry. CO_2_ reduction on reactive minerals could have converted dissolved inorganic carbon (like carbonates, Box 2) into reduced building blocks necessary for prebiotic chemical reactions. Assessing habitability on ocean worlds that may be hydrothermally active invites the same chemical questions, because interpreting the detected organics depends on inferred rather than measured boundary conditions. On Saturn’s moon Enceladus, for example, geochemical models and inferences from measurements of the moon’s plume are consistent with an alkaline, carbonate-bearing ocean^28^ and a chondritic seafloor/core that may host a range of relevant minerals (including phyllosilicates, sulfides, and carbonates)^29^. Evidence to date supports ongoing or recent water–rock interactions at elevated temperatures^30,31^ and measurements by NASA’s Cassini Mission indicate abundant reductants like H_2_, alongside complex organic material in ejected ice grains^32–34^. Because ammonia (NH_3_) was also detected by Cassini in Enceladus’ plume^35^, the demonstration of acetamide formation by Zhong et al. suggests C chemistry may also be plausible (e.g., peptide bonding of amino acids). These observations do not uniquely identify formation mechanisms, nor do they distinguish biological and abiotic generation, but they do highlight the need for interpretation and constraints. Zhong et al. offer an approach that can be tailored to more environmental or planetary contexts and be used to bracket which reduced products (e.g., CO/formate/CH_4_/C_2_) are accessible under specific redox conditions. From this, we can better interpret data from indirect observation as consistent (or inconsistent) with particular potential windows and, of particular importance in a planetary context, whether those products persist and accumulate above detection thresholds, or whether they are diluted, transformed, or consumed along transport pathways. This question matters for early-Earth interpretations and for assessing abiotic organic inventories in ocean world settings.

## Conclusion

Zhong et al. propose a testable route by which common seafloor minerals participate in abiotic carbon reduction: trace transition-metal cations adsorbed to mineral substrates become catalytically active surface sites under reducing conditions, and the surrounding mineral matrix promotes that surface chemistry^7^. Unlike Ca/Mg carbonates that evolve mostly H_2_, carbonates with adsorbed Zn shifted selectivity toward CO/formate, while those with Cu extended reduction to CH_4_/C_2_+ products (ethylene, ethanol, acetate). Excitingly, the presence of NH_3_-generated acetamide under the same potentials broadens the relevance to C–N chemistry. In the context of hydrothermal systems, these results reinforce the need to consider geo-electrochemical boundary conditions alongside pH, temperature, and bulk redox state as a predictor of product identity and yield. The implication is therefore contextual rather than universal: the mineral-supported Cu/Zn chemistry identified in Zhong et al. should matter most where carbonate/phyllosilicate substrates, trace metals, and sustained reducing potentials overlap—conditions most naturally approached in alkaline hydrothermal systems, including perhaps on ocean worlds like Enceladus.

## References

[1] Nakamura, R. et al. Electrical current generation across a black smoker chimney. *Angew. Chem. Int. Ed.* **49**, 7692–7694 (2010). 10.1002/anie.201003311 20839203

[2] Yamamoto, M. et al. Generation of electricity and illumination by an environmental fuel cell in deep-sea hydrothermal vents. *Angew. Chem. Int. Ed.* **52**, 10758–10761 (2013). 10.1002/anie.201302704 24000166

[3] Yamamoto, M. et al. Spontaneous and widespread electricity generation in natural deep-sea hydrothermal fields. *Angew. Chem. Int. Ed.* **56**, 5725–5728 (2017). 10.1002/anie.201701768 28378459

[4] Ooka, H., McGlynn, S. E. & Nakamura, R. Electrochemistry at deep-sea hydrothermal vents: utilization of the thermodynamic driving force towards the autotrophic origin of life. *ChemElectroChem* **6**, 1316–1323 (2019).

[5] Kitadai, N. et al. Geoelectrochemical CO production: Implications for the autotrophic origin of life. *Sci. Adv.* **4**, eaao7265 (2018). 29632890 10.1126/sciadv.aao7265 PMC5884689

[6] Kitadai, N. et al. Metals likely promoted protometabolism in early ocean alkaline hydrothermal systems. *Sci. Adv.* **5**, eaav7848 (2019). 31223650 10.1126/sciadv.aav7848 PMC6584212

[7] Zhong, Y. et al. Abiotic CO_2_ reduction promoted by carbonate and phyllosilicate minerals on the primitive seafloor. *Nat. Commun.* (2026). 10.1038/s41467-026-71130-7 PMC13065920 41957011

[8] Stefánsson, A. & Arnórsson, S. Gas pressures and redox reactions in geothermal fluids in Iceland. *Chem. Geol.* **190**, 251–271 (2002).

[9] Becking, L. G. M. B., Kaplan, I. R. & Moore, D. Limits of the natural environment in terms of pH and oxidation-reduction potentials. * J. Geol.* **68**, 243–284 (1960).

[10] Lee, H.-E. et al. Osmotic energy conversion in serpentinite-hosted deep-sea hydrothermal vents. *Nat. Commun.* **15**, 8193 (2024). 39322632 10.1038/s41467-024-52332-3 PMC11424637

[11] Barge, L. M., Krause, F. C., Jones, J.-P., Billings, K. & Sobron, P. Geoelectrodes and fuel cells for simulating hydrothermal vent environments. *Astrobiology* **18**, 1147–1158 (2018). 30106308 10.1089/ast.2017.1707

[12] Barge, L. M. et al. From chemical gardens to fuel cells: generation of electrical potential and current across self-assembling iron mineral membranes. *Angew. Chem. Int. Ed.* **54**, 8184–8187 (2015). 10.1002/anie.201501663 25968422

[13] Hori, Y., Wakebe, H., Tsukamoto, T. & Koga, O. Electrocatalytic process of CO selectivity in electrochemical reduction of CO_2_ at metal electrodes in aqueous media. *Electrochim. Acta* **39**, 1833–1839 (1994).

[14] Seim, I. K., Chhetri, M., Jones, J.-P. & Yang, M. Engineering intricacies of implementing single-atom alloy catalysts for low-temperature electrocatalytic CO2 reduction. *Chem. Catal.* **4**, 101164 (2024).

[15] Roldan, A. et al. Bio-inspired CO_2_ conversion by iron sulfide catalysts under sustainable conditions. *Chem. Commun.* **51**, 7501–7504 (2015). 10.1039/c5cc02078f 25835242

[16] Hudson, R. et al. CO_2_ reduction driven by a pH gradient. *Proc. Natl. Acad. Sci. USA* **117**, 22873–22879 (2020). 32900930 10.1073/pnas.2002659117 PMC7502746

[17] Lee, J.-E. et al. In situ FTIR study of CO_2_ reduction on inorganic analogues of carbon monoxide dehydrogenase. *Chem. Commun.* **57**, 3267–3270 (2021). 10.1039/d0cc07318k 33650585

[18] Yamaguchi, A. et al. Electrochemical CO_2_ reduction by Ni-containing iron sulfides: how is CO_2_ electrochemically reduced at bisulfide-bearing deep-sea hydrothermal precipitates?. *Electrochim. Acta* **141**, 311–318 (2014).

[19] Yamaguchi, A. et al. Multi-regression analysis of CO_2_ electroreduction activities on metal sulfides. *J. Phys. Chem. C* **126**, 2772–2779 (2022).

[20] Konn, C., Charlou, J. L., Holm, N. G. & Mousis, O. The production of methane, hydrogen, and organic compounds in ultramafic-hosted hydrothermal vents of the mid-Atlantic ridge. *Astrobiology* **15**, 381–399 (2015). 25984920 10.1089/ast.2014.1198 PMC4442600

[21] Lang, S. Q., Butterfield, D. A., Schulte, M., Kelley, D. S. & Lilley, M. D. Elevated concentrations of formate, acetate and dissolved organic carbon found at the Lost City hydrothermal field. *Geochim. Cosmochim. Acta* **74**, 941–952 (2010).

[22] Hemley, J. J., Cygan, G. L., Fein, J. B., Robinson, G. R. & d’Angelo, W. M. Hydrothermal ore-forming processes in the light of studies in rock-buffered systems; I, Iron-copper-zinc-lead sulfide solubility relations. *Econ. Geol.* **87**, 1–22 (1992).

[23] Metz, S. & Trefry, J. H. Chemical and mineralogical influences on concentrations of trace metals in hydrothermal fluids. *Geochim. Cosmochim. Acta* **64**, 2267–2279 (2000).

[24] Sander, S. G., Koschinsky, A., Massoth, G., Stott, M. & Hunter, K. A. Organic complexation of copper in deep-sea hydrothermal vent systems. *Environ. Chem.* **4**, 81–89 (2007).

[25] Hsu-Kim, H., Mullaugh, K. M., Tsang, J. J., Yucel, M. & Luther, G. W. Formation of Zn- and Fe-sulfides near hydrothermal vents at the Eastern Lau Spreading Center: implications for sulfide bioavailability to chemoautotrophs. *Geochem Trans.* **9**, 6 (2008). 18489753 10.1186/1467-4866-9-6 PMC2396607

[26] Estes, E. R. et al. Differential behavior of metal sulfides in hydrothermal plumes and diffuse flows. *ACS Earth Space Chem.* **6**, 1429–1442 (2022).

[27] Stüeken, E. E. Hydrothermal vents and organic ligands sustained the Precambrian copper budget. *Geochem. Persp. Lett*. 12–16 (2020). 10.7185/geochemlet.2037

[28] Glein, C. R. & Waite, J. H. The carbonate geochemistry of Enceladus’ Ocean. *Geophys. Res. Lett.* **47**, e2019GL085885 (2020).

[29] Zolotov, M. Y. An oceanic composition on Early and today’s Enceladus. *Geophys. Res. Lett.* **34**, L23203 (2007).

[30] Hsu, H.-W. et al. Ongoing hydrothermal activities within Enceladus. *Nature* **519**, 207–210 (2015). 25762281 10.1038/nature14262

[31] Sekine, Y. et al. High-temperature water–rock interactions and hydrothermal environments in the chondrite-like core of Enceladus. *Nat. Commun.* **6**, 8604 (2015). 26506464 10.1038/ncomms9604 PMC4639802

[32] Waite, J. H. et al. Cassini finds molecular hydrogen in the Enceladus plume: Evidence for hydrothermal processes. *Science* **356**, 155–159 (2017). 28408597 10.1126/science.aai8703

[33] Postberg, F. et al. Macromolecular organic compounds from the depths of Enceladus. *Nature* **558**, 564–568 (2018). 29950623 10.1038/s41586-018-0246-4 PMC6027964

[34] Khawaja, N. et al. Detection of organic compounds in freshly ejected ice grains from Enceladus’s ocean. *Nat. Astron.* **9**, 1662–1671 (2025). 41267802 10.1038/s41550-025-02655-y PMC12626887

[35] Waite Jr, J. H. et al. Liquid water on Enceladus from observations of ammonia and 40Ar in the plume. *Nature* **460**, 487–490 (2009).

